# Design, synthesis, and structural elucidation of novel NmeNANAS inhibitors for the treatment of meningococcal infection

**DOI:** 10.1371/journal.pone.0223413

**Published:** 2019-10-16

**Authors:** Osama I. Alwassil, Sandeep Chandrashekharappa, Susanta K. Nayak, Katharigatta N. Venugopala

**Affiliations:** 1 Department of Pharmaceutical Sciences, College of Clinical Pharmacy, King Faisal University, Al-Ahsa, Kingdom of Saudi Arabia; 2 Department of Pharmaceutical Sciences, College of Pharmacy, King Saud bin Abdulaziz University for Health Sciences, Riyadh, Kingdom of Saudi Arabia; 3 Institute for Stem Cell Biology and Regenerative Medicine, NCBS, TIFR, GKVK, Bangalore, India; 4 Department of Chemistry, Visvesvaraya National Institute of Technology, Nagpur, Maharashtra, India; 5 Department of Biotechnology and Food Technology, Faculty of Applied Science, Durban University of Technology, Durban, South Africa; Management & Science University, MALAYSIA

## Abstract

*Neisseria meningitidis* is the primary cause of bacterial meningitis in many parts of the world, with considerable mortality rates among neonates and adults. In Saudi Arabia, serious outbreaks of *N*. *meningitidis* affecting several hundreds of pilgrims attending Hajj in Makkah were recorded in the 2000–2001 season. Evidence shows increased rates of bacterial resistance to penicillin and other antimicrobial agents that are used in the treatment of the meningococcal disease. The host’s immune system becomes unable to recognize the polysialic acid capsule of the resistant *N*. *meningitidis* that mimics the mammalian cell surface. The biosynthetic pathways of sialic acid (i.e., *N*-acetylneuraminic acid [NANA]) in bacteria, however, are somewhat different from those in mammals. The largest obstacle facing previously identified inhibitors of NANA synthase (NANAS) in *N*. *meningitidis* is that these inhibitors feature undesired chemical and pharmacological characteristics. To better comprehend the binding mechanism underlying these inhibitors at the catalytic site of NANAS, we performed molecular modeling studies to uncover essential structural aspects for the ultimate recognition at the catalytic site required for optimal inhibitory activity. Applying two virtual screening candidate molecules and one designed molecule showed promising structural scaffolds. Here, we report ethyl 3-benzoyl-2,7-dimethyl indolizine-1-carboxylate (INLZ) as a novel molecule with high energetic fitness scores at the catalytic site of the NmeNANAS enzyme. INLZ represents a promising scaffold for NmeNANAS enzyme inhibitors, with new prospects for further structural development and activity optimization.

## Introduction

**Fig 1:**
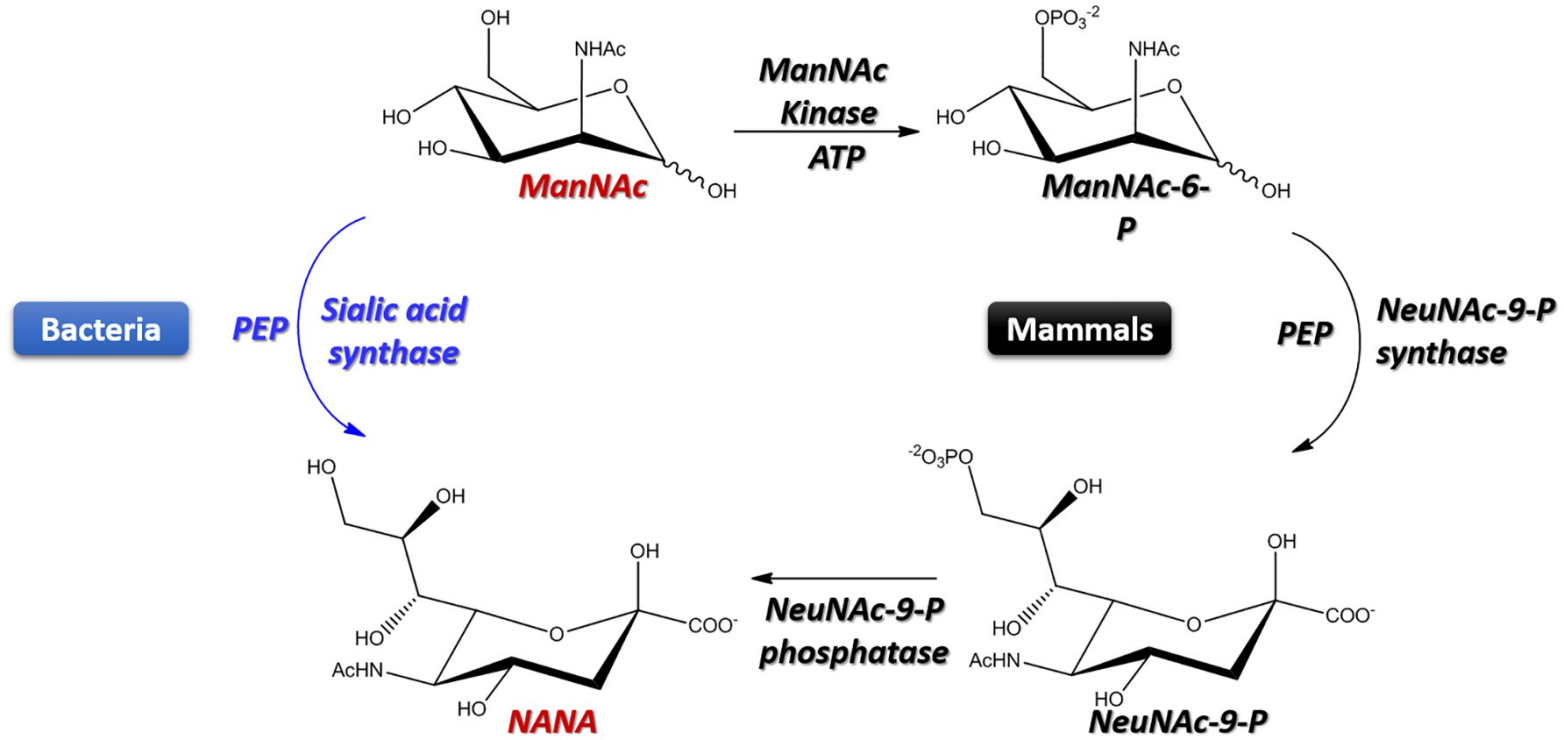
Biosynthesis of sialic acid, NeuNAc. The bacterial pathway is in blue, whereas the mammalian pathway is in black.

*Neisseria meningitidis* infection is the primary cause of bacterial meningitis infections in children (2–18 years) in the United States, with an incidence rate of about 800–1,500 people infected each year [1–4]. Reported evidence shows an increased rate of resistance to penicillin and other antibiotics currently used in the management of this disease. The mechanism of resistance to those antibiotics involves the production of altered forms of a penicillin-binding protein or altered forms of the dihydropteroate synthase enzyme [5]. Of those patients currently receiving antibiotic treatment, there is a 10%–15% mortality rate and 11%–19% of the survivors suffer from serious complications that include blindness, permanent deafness, hydrocephalic seizures, developmental delay in children, and motor skills disorders [2]. Locally, a serious outbreak of *N*. *meningitidis* affecting several hundreds of pilgrims attending Hajj in Makkah, Saudi Arabia, was recorded in the 2000–2001 season [6, 7]. Globally, endemic meningococcal infection was reported with an annual incidence rate of around 1–3 per 100,000 individuals in some countries, whereas other developing countries experienced recurrent meningococcal disease epidemics, especially in regions such as the African meningitis belt, which is considered to carry the highest burden of the disease (318,400 deaths in 2016) [1, 8, 9]. Among the most valuable strategies to overcome these challenges, the novel discovery of effective antimicrobial agents against newer bacterial targets is needed to treat resistant strains of *N*. *meningitidis*. One novel target that was found to potentially be effective against *N*. *meningitidis* is the *N*-acetylneuraminic acid synthase (Fig 1) [10–12].

*N*. *meningitidis* protects itself from the host’s immune system by synthesizing a polysialic acid capsule to mimic the mammalian cell’s surface [13, 14]. The biosynthetic pathways of sialic acid (i.e., *N*-acetylneuraminic acid [NANA]) are somewhat different in mammals and bacteria (Fig 1). The biosynthesis of NANA in bacteria lacks the *N*-acetyl mannosamine (ManNAc) phosphorylation steps of the mammalian pathway and, instead, the NANAS in *N*. *meningitidis* facilitates ManNAc and phosphoenolpyruvate (PEP) condensation to produce NANA. Thus, NANAS appears to be an attractive inhibition target in the treatment of meningococcal infection. The *N*. *meningitidis* NANAS (NmeNANAS) crystal structure has been previously identified as a domain-swapped homodimer [13]. The *N*-terminal domain of NmeNANAS provides most amino acids at the active site. The *C*-terminal domain is formed by an antifreeze protein-like (AFPL) structure that attaches to and interfers with the catalytic domain of the other monomer. NmeNANAS is related to α-keto acid synthases (e.g., 3-deoxy-D-manno-octulosonate-8-phosphate synthase). However, those enzymes lack the AFPL domain. Mutagenesis data showed that the AFPL domain has a highly conserved amino acid residue (i.e., Arg314), which plays a key role at the NmeNANAS catalytic site [13].

Penicillin appears to be the most current treatment option for meningococcal disease. Other antibiotics, including rifampin, ciprofloxacin, ceftriaxone, or azithromycin, are mostly used for prophylactic purposes [15, 16]. Meningococcal disease is characterized by rapid progression that leads to death within hours [15, 17]. For this reason, using antibiotics as chemoprophylaxis is an urgent step when preventing infection among people who are in close contact with affected patients [15–18].

*N*. *meningitidis* isolates that were resistant to penicillin were first reported in 1985 [19]. Since that time, the incidence of penicillin resistance has been increasing worldwide, where resistance reached 55% in Spain in 1996 [19, 20]. In the United States, the prevalence of penicillin resistance in *N*. *meningitidis* isolates remained low until the end of the last century. Current reports, however, showed higher resistance frequencies than previous reports [21, 22]. Other *N*. *meningitidis* isolates found around the world showed resistance to other recommended antibiotics such as rifampin, ciprofloxacin, and fluoroquinolone [19, 23–25]. Thus, although penicillin and other available antibiotics have been the agents of choice when treating meningococcal disease over many years, recent reports indicate the need for more effective treatments. Although there are no known inhibitors of sialic acid synthesis currently on the market, the molecular mechanism of action of NmeNANAS inhibitors provide a promising venue and should not interfere either with the host’s sialic acid synthesis, nor any conventional antibiotics. Furthermore, improving immune system reactivity should afford needed therapeutic synergy [26–28].

The discovery and full description of the NANAS enzyme system in comparison to the mammalian system were reported between 1958–1962 [29–32]. The *N*-acetylneuraminic acid synthase enzyme system catalyzes PEP condensation via ManNAc (bacteria) or ManNAc-6P (mammals) to yield NANA or NeuNAc-9P, respectively (Fig 1) [10]. While the human enzyme can accept mannose-6-phosphate as an alternative substrate to produce 2-keto-3-deoxy-nonulosonate-6-phosphate (KDN-6P), *N*. *meningitidis* enzymes do not accept mannose as an alternative substrate [28, 32]. Another feature of sialic acid synthases is that they are metalloenzymes that require precise coordination between divalent cation cofactors [33, 34]. For all these characteristics, NmeNANAS stand out as unique drug targets in the bacterial pathway when compared to mammalian sialic acid synthase, which uses a different substrate. Inhibition of the NmeNANAS enzyme would prevent bacterial sialic acid biosynthesis, while the mammalian pathway remains intact.

As the need for an alternative scaffold continues to exist, we adopted a ligand-based and structure-based approach to identify novel chemical entities or hits that could serve as the groundwork in the development of new inhibitors of NmeNANAS. New chemical entities with high potential inhibitory activity on NmeNANAS would have great therapeutic impacts by causing polysialic acid capsule depletion and bacterial death with the least number of possible, off-target side effects [12].

In continuation of the synthesis and characterization of substituted indolizine analogs [35] for anti-tubercular [36], cyclooxygenase enzyme inhibition (COX-2) [37, 38], larvicidal [39, 40], and anticancer [41] activities, in the present investigation, we undertake the synthesis and perform a single crystal X-ray study on ethyl 3-benzoyl-2,7-dimethyl indolizine-1-carboxylate (**INLZ**) as a NANAS inhibitor.

## Materials and methods

### General

The chemicals used in this study were purchased from Sigma-Aldrich Co. (St. Louis, MO, USA). All reactions were performed in hot-air-dried glass wares in a nitrogen atmosphere with dry solvents. The various chemical reactions were observed via thin-layer chromatography (TLC). TLC was performed on Sigma-Aldrich Silica gel (Sigma-Aldrich Co.) on aluminum foil; n-hexane and ethyl acetate (4:6) were used as solvents and were visualized with an ultraviolet (UV)-light/iodine chamber. A Büchi B-545 apparatus (Büchi Labortechnik AG, Bern, Switzerland) was used to observe melting points. Fourier-transform infrared (FT-IR) spectra were recorded (S1 Fig) on a Shimadzu FT-IR spectrometer (Shimadzu, Kyoto, Japan). Nuclear magnetic resonance (NMR; 400 MHz) spectra were recorded (S2 and S3 Figs) using a Bruker-400 spectrometer (Bruker Corporation, Billerica, MA, USA) in ambient temperatures; further, CDCl_3_ was used as a solvent. Chemical shift values were measured in *δ* ppm; TMS was used as a reference. The peak multiplicities were as follows: s, singlet; d, doublet; t, triplet; q, quartet; and m, multiplet. Liquid chromatography (LC)-mass spectrometry (MS) analysis was carried out using Agilent LC-1200 (Agilent Technologies, Santa Clara, CA, USA), coupled with a 6140 single quad mass spectrometer with ESI +ve mode, MS range: 100–2,000. In addition, elemental analysis was carried out on a Thermo Finnigan FLASH EA 1112 CHN analyzer (Thermo Finnigan LLC, San Jose, CA, USA). A single-crystal X-ray diffraction study was performed using a Bruker APEX II diffractometer (Bruker Corporation), which featured a charge-coupled device (CCD) detector using Mo Kα radiation (λ = 0.71073 Å).

### Chemistry

The synthetic scheme for the construction of ethyl 3-benzoyl-2,7-dimethyl indolizine-1-carboxylate (**INLZ**) is described in Scheme 1, and physicochemical constants are tabulated in Table 1.

**Table 1:** Synthesis of ethyl 3-benzoyl-2,7-dimethylindolizine-1-carboxylate (INLZ) using the catalyst-free microwave assisted method^a^.

Comp code ^a^^,^^b^	Mol formulae (Mol mass)	Yield (%)^c^	m.p (°C)	cLogP^d^
INLZ	C_20_H_19_NO_3_ (321)	88	123–124	5.3494

^*a*^ Reaction conditions for **INLZ**: 4-Methyl pyridine (1.07 mmol), phenacyl bromide (1.07 mmol), ethyl butynoate (1.07 mmol), triethylamine (1.07 mmol), and acetonitrile (4 mL) in an 8 mL microwave tube in a nitrogen atmosphere.

^*b*^ The products were characterized using both spectral and physical data.

^*c*^ Yield after being purified via the recrystallization method.

^*d*^ *c*Log*P* was calculated using the ChemDraw Professional 16 software package.

**Scheme 1**: Microwave-assisted synthesis of ethyl 3-benzoyl-2,7-dimethyl indolizine-1-carboxylate (**INLZ**).

#### Synthesis of ethyl 3-benzoyl-2,7-dimethyl indolizine-1-carboxylate (INLZ)

A solution of triethylamine (0.108 g, 1.07 mmol) in acetonitrile (4 mL) was added to a mixture of 4-methyl pyridine (0.1 g, 1.07 mmol), phenacyl bromide (0.249 g, 1.07 mmol), and ethyl butanoate (0.120 g, 1.07 mmol) in an 8 mL capacity microwave tube under a nitrogen atmosphere. The reaction mixture was irradiated at 100°C in a microwave initiator for 5 minutes. The reaction was monitored using TLC and the reaction medium was evaporated under reduced pressure; the crude reaction mass was diluted using water, while the aqueous layer was twice extracted with ethyl acetate. The solution was then washed with brine. The residue was obtained by evaporating the organic layer under reduced pressure; the residue was purified via column chromatography (60–120 mesh silica gel) with hexane and ethyl acetate as a solvent system. This resulted in 0.343 g of **INLZ** at 88% yield. Yellow crystalline compound; FT-IR (KBr, cm^–1^): 2,979.82, 1,693.38, 1,604.66, 1,523.66, 1,342.36, 1,180.36; ^1^H-NMR (400 MHz CDCl_3_) δ = 9.47–9.45 (1H, d, *J* = 7.2 Hz), 8.16 (1H, s), 7.70–7.68 (2H, m), 7.59–7.47 (3H, m), 6.83–6.81 (1H, d, *J* = 7.2 Hz), 4.43–4.38 (2H, q, *J* = 7.2 Hz), 2.48 (3H, s), 2.20 (3H, s), 1.45–1.42 (3H, t, *J* = 7.2 Hz); ^13^C-NMR (100 MHz CDCl_3_) δ = 187.54, 165.20, 141.34, 140.11, 138.60, 138.19, 131.65, 128.79, 128.52, 127.58, 122.44, 118.04, 116.78, 104.32, 59.69, 21.63, 15.00, 14.79; LC-MS (ESI, Positive): m/z: (M+H)^+^:322; Anal. calculated for: C_20_H_19_NO_3_; C, 74.75; H, 5.96; N, 4.36; Found: C, 74.69; H, 5.99; N, 4.33.

### Crystallography

Single crystals of INLZ were obtained from a mixture of a 1:1 ratio of acetone and ethanol solvents using a slow evaporation method in ambient temperature. Single-crystal X-ray diffraction data for R13 were collected using a Bruker KAPPA APEX II DUO diffractometer with Mo-Kα radiation (χ = 0.71073 Å). Data collection was performed when the temperature reached 153(2) K. The temperature was maintained using an Oxford Cryostream cooling system (Oxford Cryostat; Oxford Cryosystems, Witney, UK). SAINT was used to perform cell refinement and data reduction [42]. Further, SADABS were used to scale the data and perform absorption correction, while SHELXS-97 was used to solve the structure [43]. Moreover, the data were scaled and further refined using a full-matrix, least-squares methods using SHELXL-2014, as based on F^2^ [43]. The non-hydrogen atoms were anisotropically refined, while the hydrogen atoms were refined using riding models, where Uiso assigned 1.2 or 1.5 times Ueq of their parent atoms and the C–H bond distances were maintained at ranges that spanned from 0.95 Å–0.99 Å. Furthermore, the structure of these atoms was refined to an R factor of 0.040. Table 2 details the crystallographic details. Finally, ORTEP and Mercury 3.8 were used to generate intermolecular interactions, a thermal ellipsoid diagram, and packing diagrams [44] [45].

**Table 2:** Crystal and measurement data for the ethyl 3-benzoyl-2,7-dimethyl indolizine-1-carboxylate (INLZ) compound.

Crystal data	Compound INLZ
Formula	C_20_H_19_NO_3_
CCDC number	1816009
Formula weight	321.36
Crystal morphology	Plate
Crystal size (mm)	0.08 × 0.15 × 0.60
Temperature/K	173(2)
Radiation	Mo K_α_
Wavelength (Å)	0.71073
Crystal system	Monoclinic
Space group	P 2_1_/c
a (Å)	15.2584(13)
b(Å)	5.2486(4)
c (Å)	21.0141(19)
α(°)	90
β(°)	106.349(2)
γ(°)	90
Volume (Å^3^)	1614.9(1)
Z	4
Density (gm/cm^3^)	1.32
μ (1/mm)	0.089
F (000)	680
θ (min, max)	1.4, 28
Total number of refl^n^	27,228
No. of unique refl^n^	3,884
No. of parameters	220
R_ obs, wR_2__obs	0.040, 0.053
Δρ_min_(eÅ^-3^), Δρ_max_(eÅ^-3^)	-0.267, 0.226
GooF	1.06

## Results and discussion

### Structural and conformational analysis of the inhibitors

**Fig 2:**
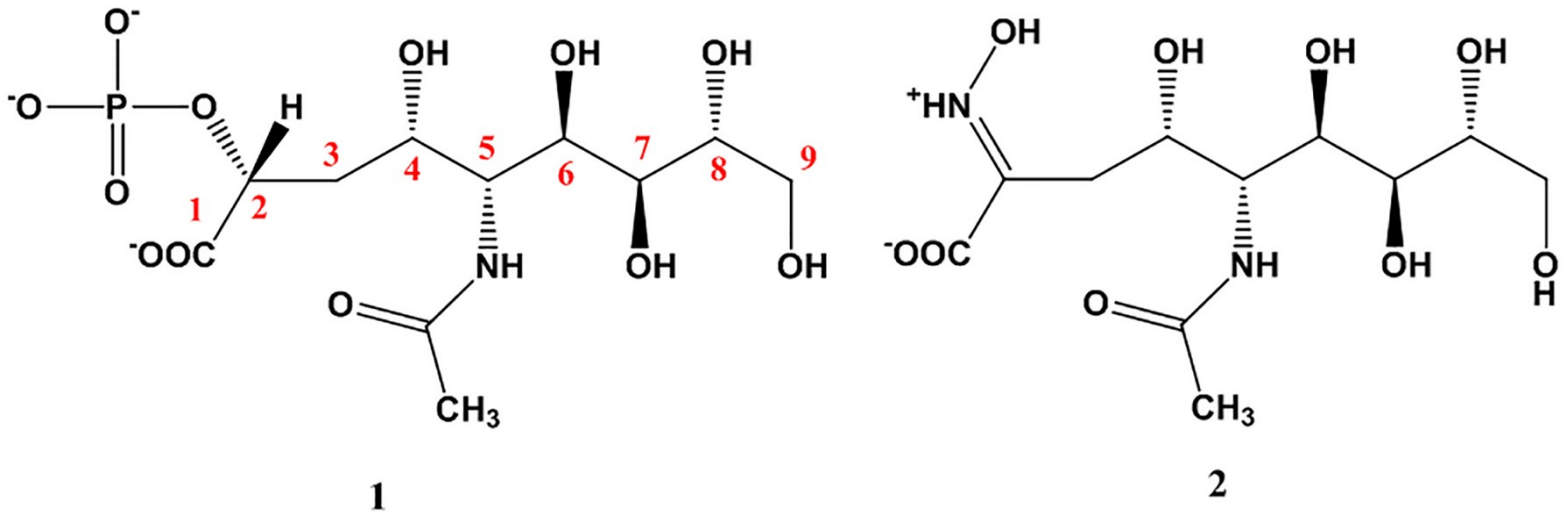
Chemical structures of known NmeNANAS inhibitors. NAMA (**1**) and NAMA oxime (**2**).

There are several known inhibitors that target NmeNANAS’s active site. Structural characterization of these inhibitors was based on NMR and X-ray crystallography studies [46]. The first published inhibitor of NmeNANAS, **1**, is a tetrahedral intermediate analog inhibitor with a *K*_i_ = 3.1 (±0.1) μM (Fig 2) [34].

**Fig 3:**
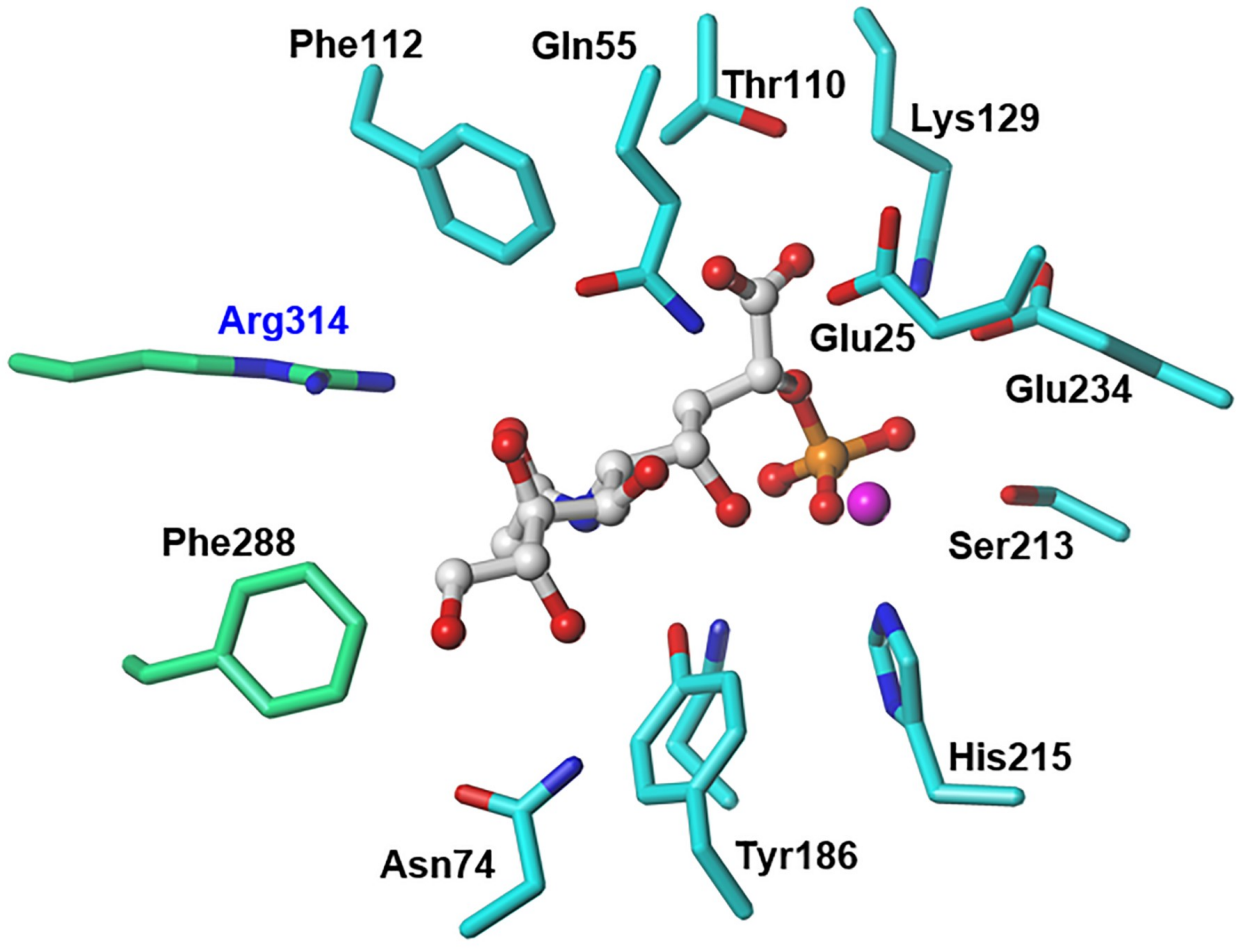
NmeNANAS catalytic site architecture. Inhibitor **1** (white) is bound by key amino acid residues from the *N*-terminal domain of a monomer (gray) and from the *C*-terminal domain of the other monomer (green). Hydrogen bonds are shown as yellow dashes.

The crystal structure of this inhibitor in the NmeNANAS catalytic site shows a preference for a (2*R*)-configuration (Figs 2 and 3) [33, 34, 46]. This finding suggests that the tetrahedral intermediate of the substrate (i.e., formed by a PEP nucleophilic attack on ManNAc) has the same configuration. Further mechanistic studies on NmeNANAS-substrate complexes indicate that the nucleophilic attack of PEP occurs from the *si* side on the *si* side of the CO double bond of ManNAc, and that Mn^2+^ is essential for optimum orientations of the two molecules [34, 47].

Another previously developed inhibitor is the NANA oxime (**2**), with a *K*_i_ = 1.6 (±0.7) pM (Fig 2) [48]. This inhibitor was developed to mimic the oxacarbenium ion, which is a less stable intermediate than the tetrahedral intermediate. The crystal structure of the NmeNANA catalytic site in the presence of NANA oxime **2** shows that the inhibitor exists in the *trans* conformation that matches the reported orientation of the natural substrates, ManNAc and PEP [34, 48]. However, both inhibitors **1** and **2** are associated with undesired characteristics. Inhibitor **1** shows partial occupancy of the Mn^2+^ site in the enzyme-inhibitor complex that might somewhat impair binding, whereas inhibitor **2** is associated with a residual enzyme activity of around 10% [34, 48]. Besides, both inhibitors violate Lipinski’s rule of five [49], a basic rule that is used to evaluate drug-likeness, as its calculated Log*P* values are beyond the acceptable lipophilicity/hydrophilicity scale that specifically characterizes druggable chemical entities.

The structural features in inhibitor **1** that allow binding to the active site and inhibit its activity have been considered in the initial setting of our approach. We consider a position analogous to one of the oxygen atoms in the phosphate functionality attached to carbon number 2 to be a critical atom that would enhance a favorable interaction energy with the Mn^2+^ ion. A molecule at its lowest energy conformation occupying the space by an electron-rich moiety should have an essential element that competes for the catalytic site of NmeNANA.

### NANAS multiple-sequence alignment studies

There are two major homologs of NmeNANAS that facilitate similar reactions: 2-keto-3-deoxy-D-arabinoheptulosonate-7-phosphate (i.e., DAH7P or AROG) synthase, and 2-keto-3-deoxy-D-mannoctulosonate-8-phosphate (i.e., KDO8P or KDSA) synthase. DAH7P synthesis is the initial step in the shikimate pathway that exists in microorganisms and plants, and is involved in the biosynthesis of aromatic amino acids [13, 46]. DAH7P synthase catalyzes the PEP and D-erythrose-4-phosphate condensation to form DAH7P. KDO8P is an important part of the innermost region of Gram-negative lipopolysaccharides and is formed by the condensation of PEP and D-arabinose-5-phosphate through the catalytic process of the KDO8P synthase enzyme [50, 51]. Despite the similarity in the catalytic mechanism and function of these homologues, there is <10% sequence identity between NmeNANAS and DAH7P/KDO8P synthases [51].

**Fig 4:**
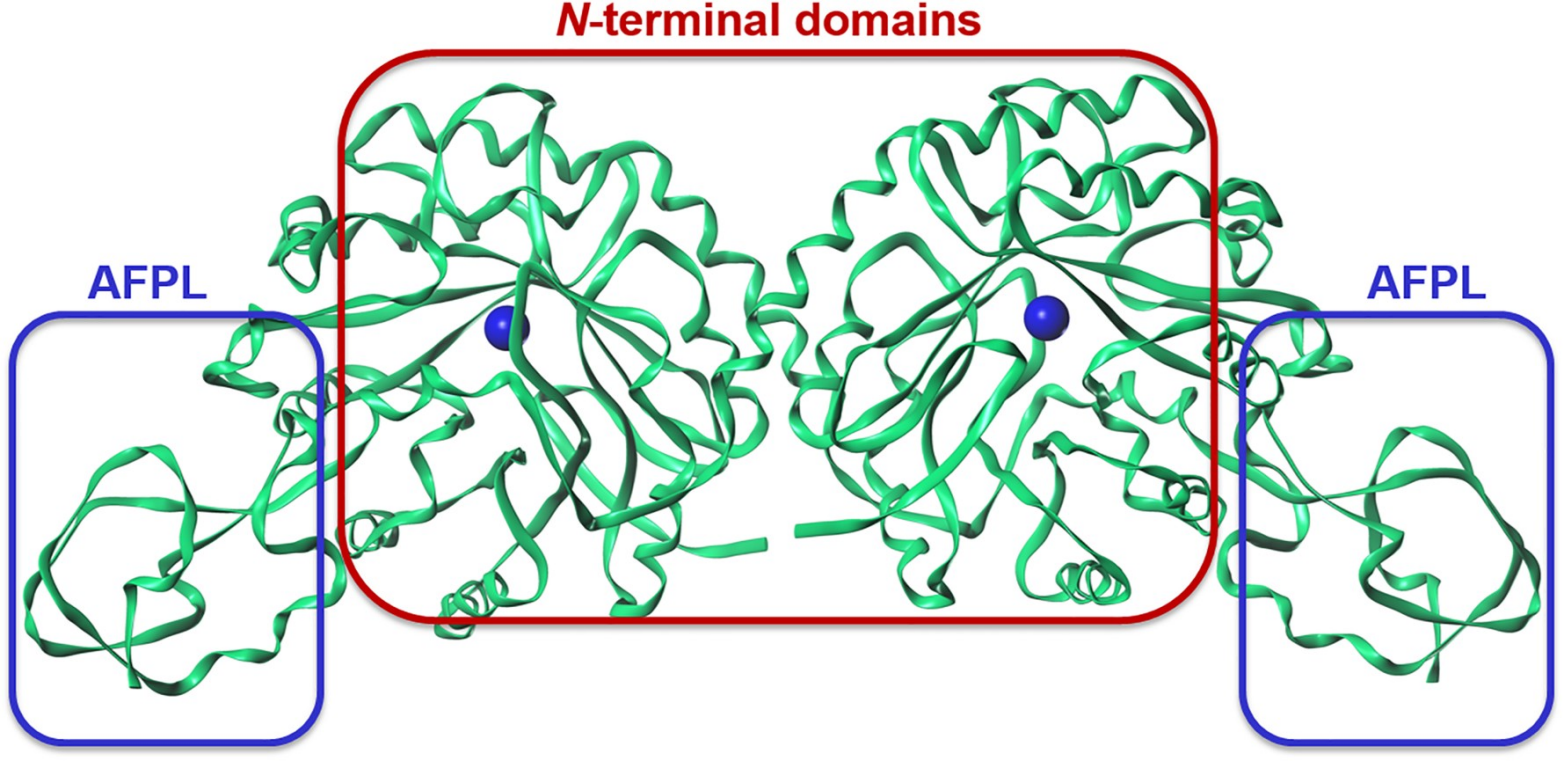
Structural arrangement of NmeNANAS as a domain-swapped homodimer. Ribbons represents the secondary structure elements. The red box represents the *N*-terminal domains, whereas the blue boxes represent the AFPL domains in the dimeric complex.

To determine the possibilities of local sequence similarity at the catalytic site, we performed multiple alignments of the NmeNANAS sequence and for the sequences obtained for DAH7P synthase and KDO8P synthase. The catalytic sites of the homologs shared a very low percentage of identity and only one amino acid of the 13 important residues was found to be an identical match (Fig 4).

### NANAS structural characterization

**Fig 5:**
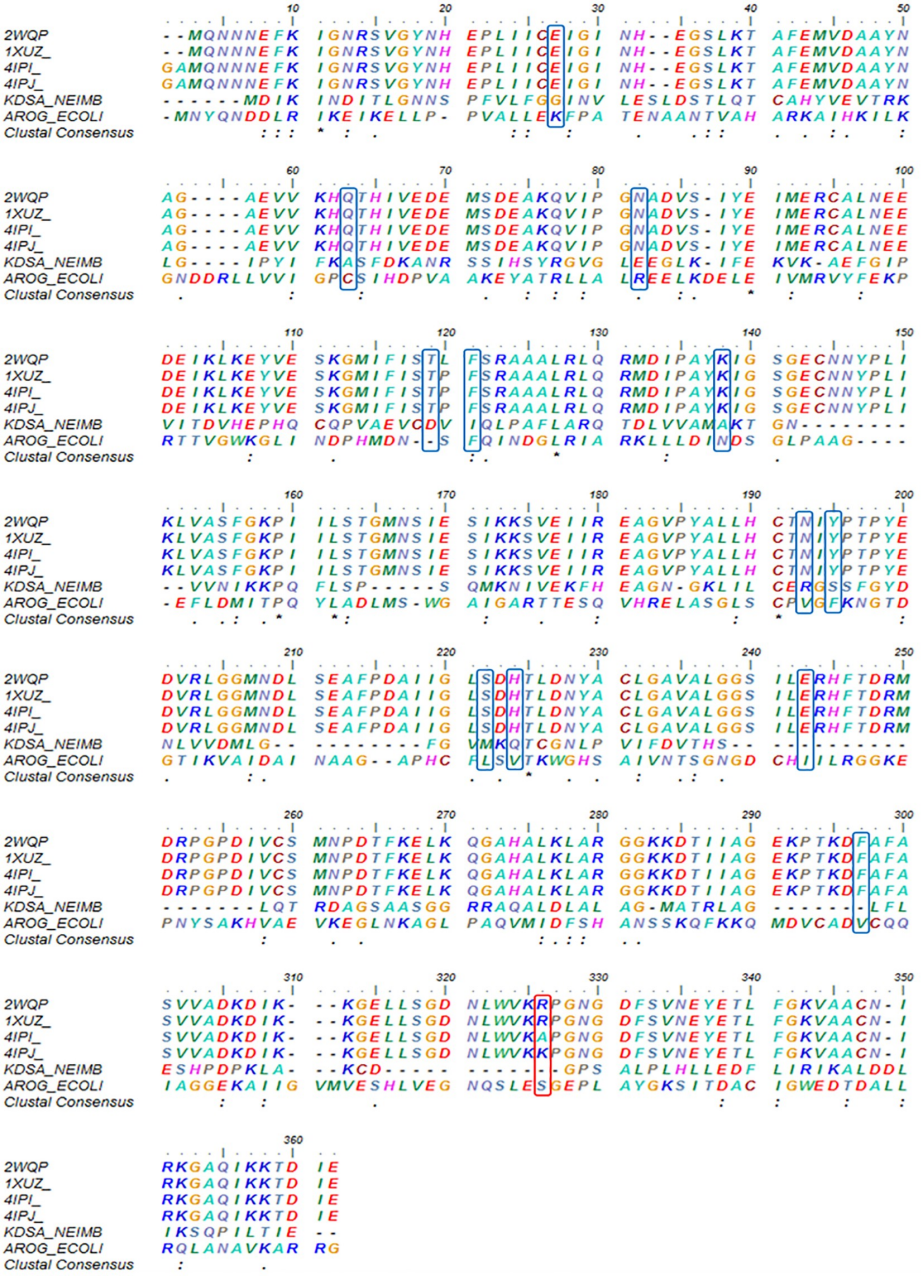
Multiple-sequence alignment of six amino acid sequences. The first four are the available crystal structures of NANAS, 2-dehydro-3-deoxyphosphooctonate aldolase of *N*. *meningitidis*, and Phospho-2-dehydro-3-deoxyheptonate aldolase of *Escherichia coli*. Alignments show a low degree of overlapping identity between the NANAS amino acid sequence and enzymes catalyzing similar reactions. The asterisks (*) indicate conserved amino acids, whereas the colons (:) and periods (.) indicate strongly and weakly conserved amino acids, respectively. The blue boxes indicate important amino acid residues lining the binding site from the N-terminal domain, while the red box indicates the amino acid lining the binding site from the AFPL domain.

There are seven available crystal structures of *N*. *meningitidis* NANAS (NmeNANAS) in the protein data bank (i.e., 1XUZ, 1XUU, 2ZDR, 3CM4, 2WQP, 4IPI, and 4IPJ) [13, 33, 34, 52]. The shape of the binding pockets in those crystal structures is only similar in the area around the catalytic metal. Only one crystal structure (i.e., 2WQP) of NmeNANAS is in complex with an inhibitor (Figs 2 and 4). This crystalized inhibitor mimics the tetrahedral intermediate formed in the normal NmeNANAS reaction [33]. Structurally, the NmeNANAS enzyme is a domain-swapped homodimer (Fig 4). The *N*-terminal domain of NmeNANAS forms an (α/β)_8_ barrel shape and contains most of the amino acids at the active site, whereas the *C*-terminal domain forms an AFPL domain capping the catalytic domain of the other monomer [13]. The AFPL domain of NmeNANAS is unique and has not been observed in similar homologs (Fig 5). Previously reported mutagenesis data by Joseph et al. showed that the AFPL domain has a highly conserved amino acid residue (i.e., Arg314; Figs 3, 4 and 5), which plays a key role in the NmeNANAS catalytic site [13]. Arg314 is the only residue of the AFPL domain to contribute to the active site of NmeNANAS in a direct manner. The detailed role of Arg314 in the NmeNANAS system was investigated by mutagenesis studies.

In addition to mutagenesis studies, the role of Arg314 in NmeNANAS was previously investigated via molecular modeling and isothermal titration calorimetry [13]. The amino acid, Arg314, was substituted by Lys to reduce the hydrogen-bonding capabilities of the side chain while maintaining some functionality of the wild-type protein. Another mutant enzyme was introduced by substituting Arg314 by Ala, which completely eliminates the ability of the side chain to foster hydrogen bonds at this residue [13]. Arg314 and ManNAc can exhibit tow primary interactions in the wild-type enzyme, and these might result in the accurate placement of *N*-acetyl, and thus the aldehyde group. An Arg to Ala mutation in R314A NmeNANAS can lead negatively impact the hydrogen bond and electrostatic interactions between the Arg314 residue and ManNAc. Conversely, an Arg to Lys mutation in R314K NmeNANAS can retain the positive charge on the functionality of the side chain, but it may also result in a loss of hydrogen bonds. The observation that R314K NmeNANAS demonstrates catalytic activity, while R314A NmeNANAS does not, indicates that Arg plays an essential and indirect role in catalysis [13].

### Preparations of the virtual target

The X-ray crystal structure of the NmeNANAS enzyme in complex with inhibitor **1** (PDB ID: 2WQP) was used to identify the important molecular interactions involved in this association. Hydrogen atoms were added and subjected to a minimization process with the Tripos force field (Gasteiger-Hückel charges; distance-dependent dielectric constant = 4.0), while heavy atoms remained untouched. The task here was to identify the desirable features in inhibitor **1** that allowed binding to the active site and inhibited its activity, while considering the important amino acids lining the catalytic site. We found that Glu25, Gln55, Asn74, Thr110, Phe112, Lys129, Asn184, Tyr186, Ser213, His215, Glu234, Phe288, and Arg314 amino acids are in close proximity and have essential interactions with the ligand. We used computational molecular discovery methods to identify compounds that may have sets of chemical features that could allow for an association with the NmeNANAS enzyme. Those features are believed to be necessary for recognition by the enzyme and, consequently, allow for the desired inhibition.

### Virtual screening

A structure-based, drug-design approach depends on the amount of knowledge about the three-dimensional structure of the associated region of biomolecular complexes. On the other hand, a ligand-based drug design approach depends on the amount of knowledge about the physicochemical properties of ligands or substrates that allow association to their molecular targets. Here, we combine the two methods and we used a ligand and structure-based drug design approach to advance the quality/quantity of desired hits. The combined knowledge of the association enabled us to construct queries and apply them to virtual libraries of compounds using UNITY. The hits from the UNITY module search were visually observed within SYBYL-X 2.1.1 [53] to identify molecules that were drug-like; these were then used to scan the databases of diverse chemical compounds, where we searched for analogous association features and their three-dimensional relationships from the query to the compounds in the database. The virtual libraries of compounds used (i.e., the National Cancer Institute [NCI], Maybridge [Thermo Fisher Scientific, Inc.], and ZINC databases) [54] included a fairly large number of compounds. Our first defined query point is a “negative center”, sphere of 1 Å radius, near the catalytic metal in a position analogous to one of the oxygen atoms in the phosphate functionality attached to carbon number 2 of inhibitor **1**. Secondly, a “donor atom” point was defined at one of the terminal nitrogen atoms of the guanidinium moiety, sphere of 0.5 Å radius, of Arg314 residue. The third defined point is the “donor atom” spot of the terminal oxygen atom, with a sphere of 0.5 Å radius, of the Thr110 amino acid. A fourth “aromatic hydrophobic” core center, sphere of 1.5 Å radius, was defined in the vicinity of the aromatic rings of two important amino acid residues (i.e., Tyr186 and Phe288; Table 3). The number of candidate compounds of those query points was ~16,200. This number was reduced to a more manageable number of 43 compounds by adding one critical query point: a “donor atom” spot defined at the terminal nitrogen position, sphere of 0.5 Å radius, of the Asn74 residue. Receptor-site constraints were positioned around heavy atoms of the amino acids surrounding the active site pocket. Further assessment was performed by subjecting the final candidate compounds to the docking process inside the active site of NmeNANAS using the genetic algorithm-based, ligand-docking program, GOLD [55]. To determine the most likely binding mode of a candidate compound, the HINT (Hydropathic INTeractions) [55] scoring function was used to rank the GOLD docking poses by analyzing the structural aspects of the interactions. In contrast to the default software scoring function, the HINT score can be correlated with the binding free energy [55]. In addition, visual analysis of the binding modes of the top scored solutions was considered to ensure that significant interactions were maintained with the essential amino acid residues of the catalytic site.

**Table 3:** Summary of the key interacting amino acid residues at the catalytic site of NmeNANAS.

Enzyme domain	*N*-terminal domain	C-terminal domain
**Amino acid residues**	Glu25, Gln55, Asn74, Thr110, Phe112, Lys129, Asn184, Tyr186, Ser213, His215, Glu234, Phe288	Arg314

### Lead compounds

**Fig 6:**
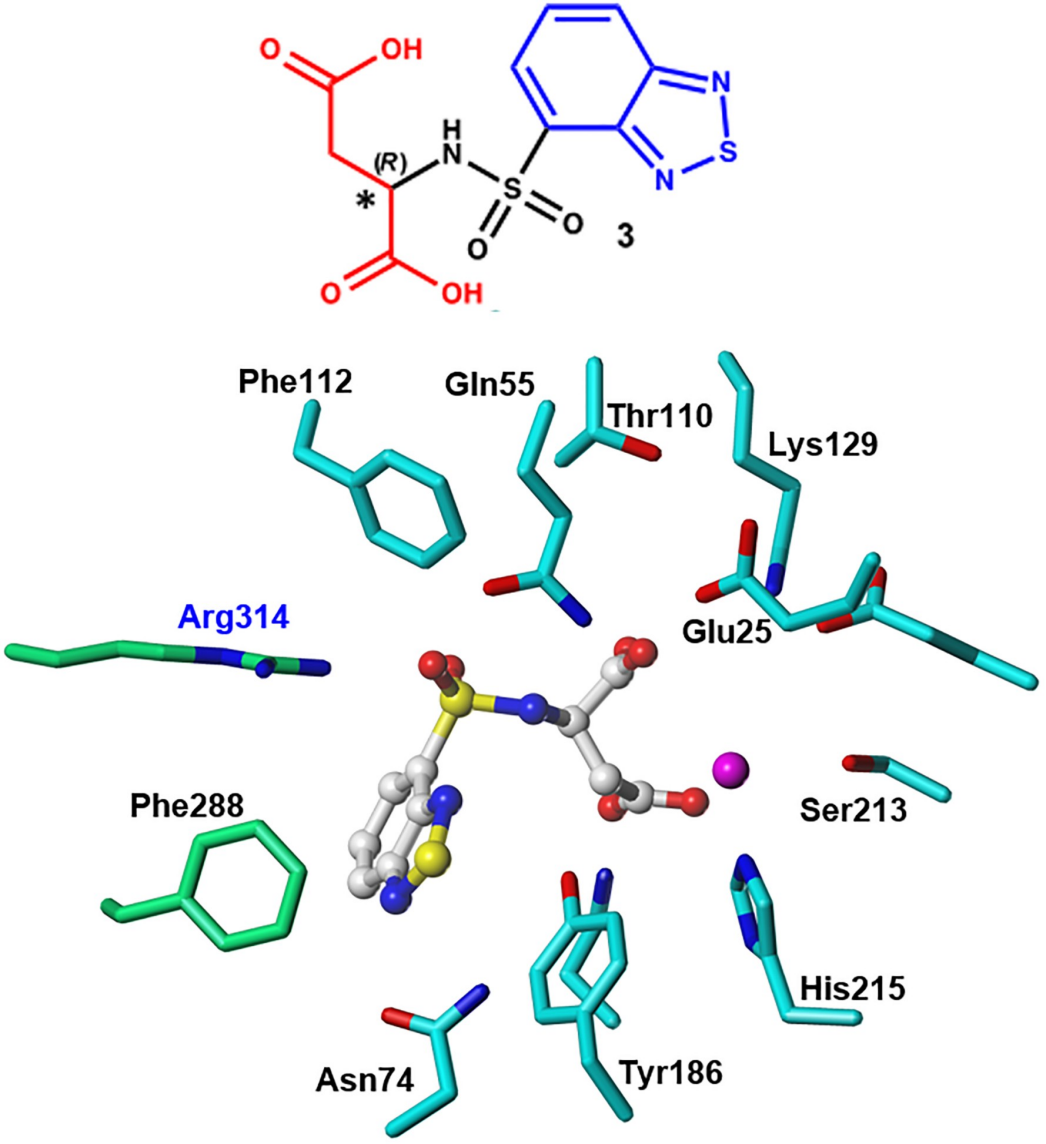
Representative structure of ZINC02337049 (3) and its interaction mode with the NmeNANAS catalytic site.

**Fig 7:**
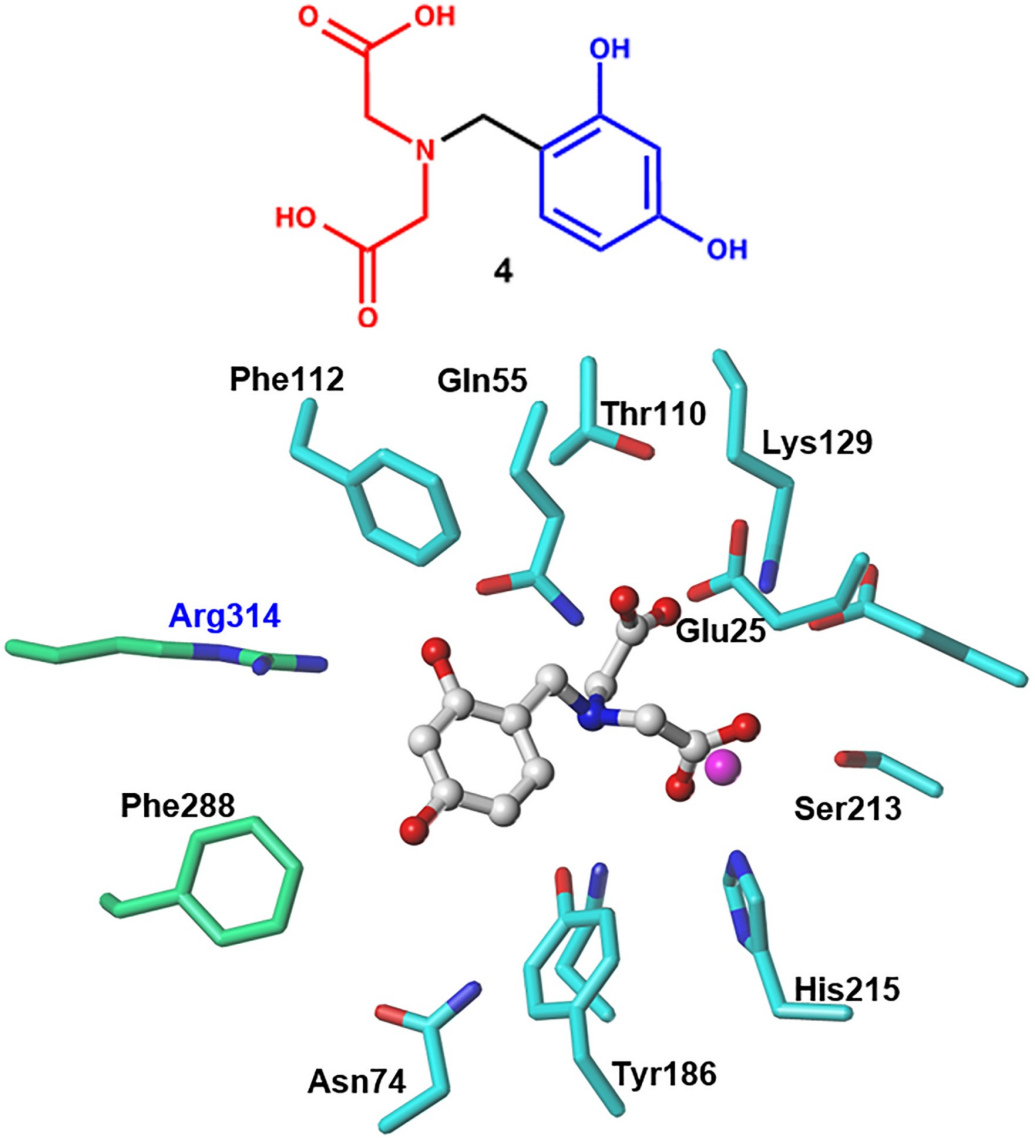
Representative structure of ZINC00968695 (4) and its interaction mode with the NmeNANAS catalytic site.

By examining a diverse set of compounds, we searched for new chemical entities that possessed sufficient interactions at the binding pocket of the NmeNANAS enzyme. However, for the purposes of this study, we selected two candidate molecules for further structural studies. The two selected molecules were found to have significant interactions with the surrounding residues in the catalytic region, and they had higher CHEMPLP and HINT scores when compared to the rest of the docked compounds. These two molecules are **ZINC02337049** (i.e., **3**; Fig 6) and **ZINC00968695** (i.e., **4**; Fig 7), and their CHEMPLP scores were 58 and 60, respectively. HINT scoring for **ZINC02337049** was 4,849, while it was 4,776 for **ZINC00968695**. Both molecules complied with Lipinski’s rule of five [33] (e.g., the calculated log *P* values for **ZINC02337049** and **ZINC00968695** were –0.891 and –1.654, respectively). The fundamental goal was to reach the highest possible inhibition effect on NmeNANAS by identifying a more active analog. The analog with a higher affinity was assumed to possess more molecular interactions, and vice versa. An effective way to achieve this, both in terms of economics and time, was to conduct an initial structure–activity relationship (SAR) study that could be used in additional advanced structural studies in the future.

Visual analysis of the interaction mode of **ZINC02337049** showed a bidentate interaction of the sulfonyl group with the Arg314 residue. One carboxylate group forms a hydrogen bond with Thr110 amino acid residue, while the other appears to exist in the vicinity of the catalytic metal. The aromatic portion adopts an edge-to-face π-π interaction with the Phe288 residue (Fig 6).

The compound with the second-best score compound, **ZINC00968695**, showed similar interactions for the terminal carboxylate groups. The protonated amine center formed a hydrogen bond with the Asn184 amino acid residue. The two hydroxyl groups on the aromatic rings formed a hydrogen bond with Asn74 and Arg314 residues, while the aromatic ring appeared to adopt an edge-to-face π-π interaction with Phe288 and Tyr186 (Fig 7). In comparison with **ZINC02337049**, the interactions of the binding pocket of NANAS in both situations appeared to have involved similar interactions with certain amino acids.

### Designed molecule

**Fig 8:**
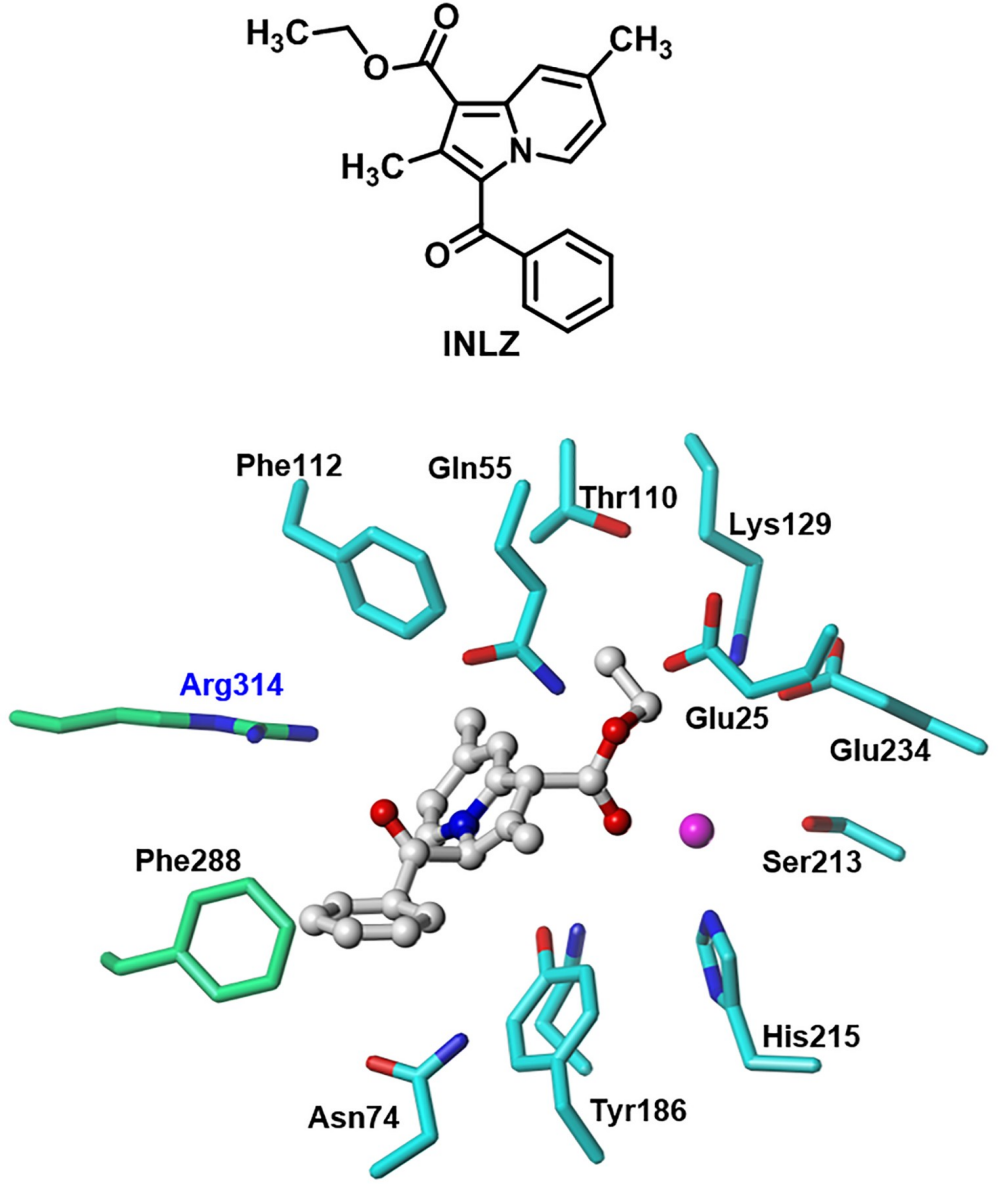
Designed structure of ethyl 3-benzoyl-2,7-dimethylindolizine-1-carboxylate (INLZ) and its interaction mode with the NmeNANAS catalytic site.

Based on the above analysis, taking advantage of this information may be necessary to obtain a molecule with potential inhibitory effects. The designed molecule, ethyl 3-benzoyl-2,7-dimethyl indolizine-1-carboxylate (**INLZ**), complied with Lipinski’s rule of five [33] (e.g., its calculated log *P* value was 4.64). **INLZ** shows higher CHEMPLP scores (64) compared to both **ZINC02337049** and **ZINC00968695** molecules which scored (58) and (60), respectively. Hydropathic analysis of HINT for **INLZ** indicated a slight improvement, with a score of 5,079. These results linked directly to the structural variations of the three candidate molecules, as well as to the extent of polar and hydrophobic moieties within them. **INLZ** maintained important polar interactions with the Mn^2+^ ion, as well as with the Gln55, Asn184, and Arg314 residues, whereas the aromatic rings appeared to have played an increased role in the edge-to-face π-π interactions with Phe112, Tyr186, and Phe288 (Fig 8).

### Single-crystal X-ray study

**Fig 9:**
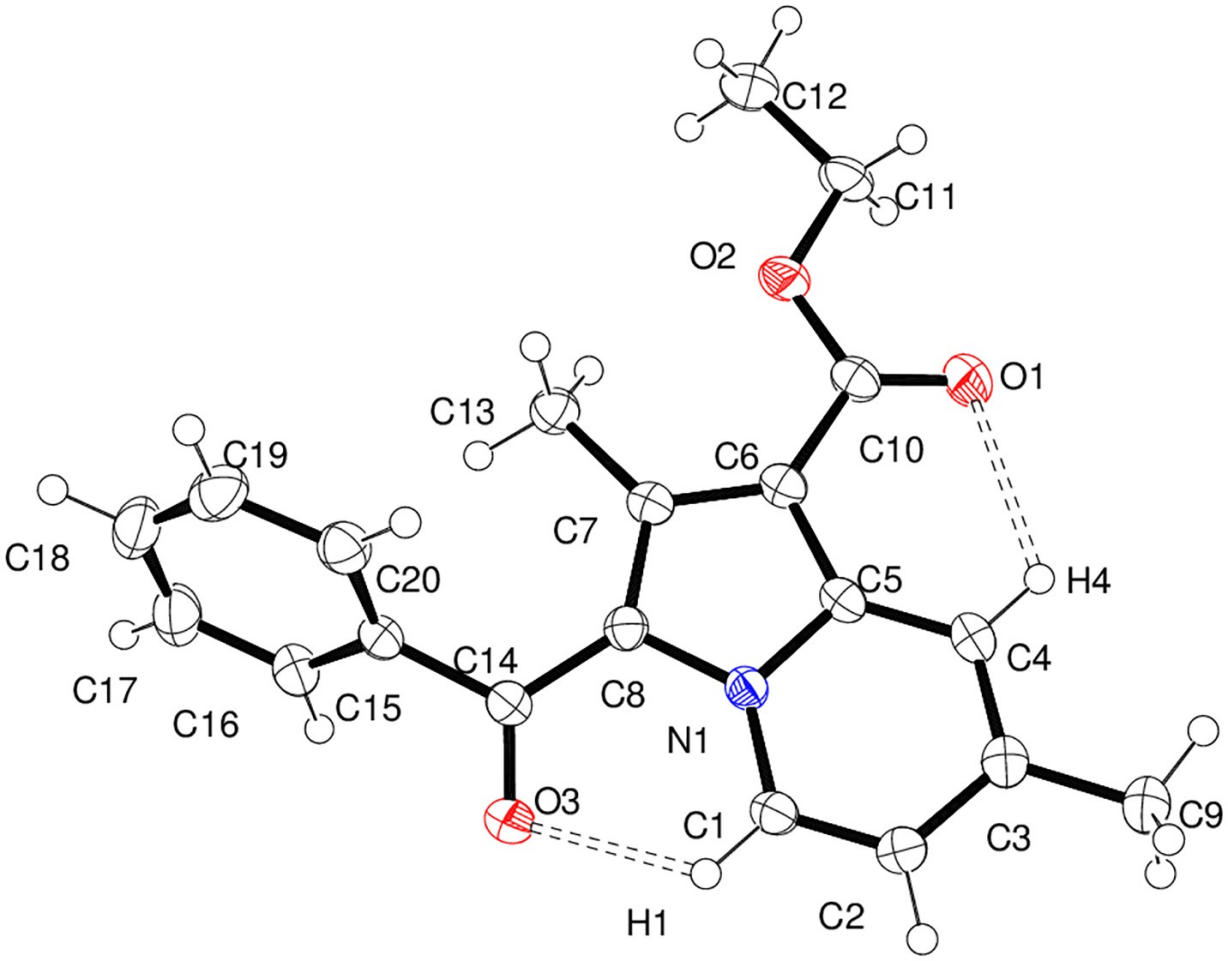
Thermal ellipsoid 50% probability plot with atom labelling. The molecule adopts the conformation with C-H⋯O intra-molecular interactions, as shown in dotted lines. For clarity, the non-participating hydrogen atoms are not labeled.

**Fig 10:**
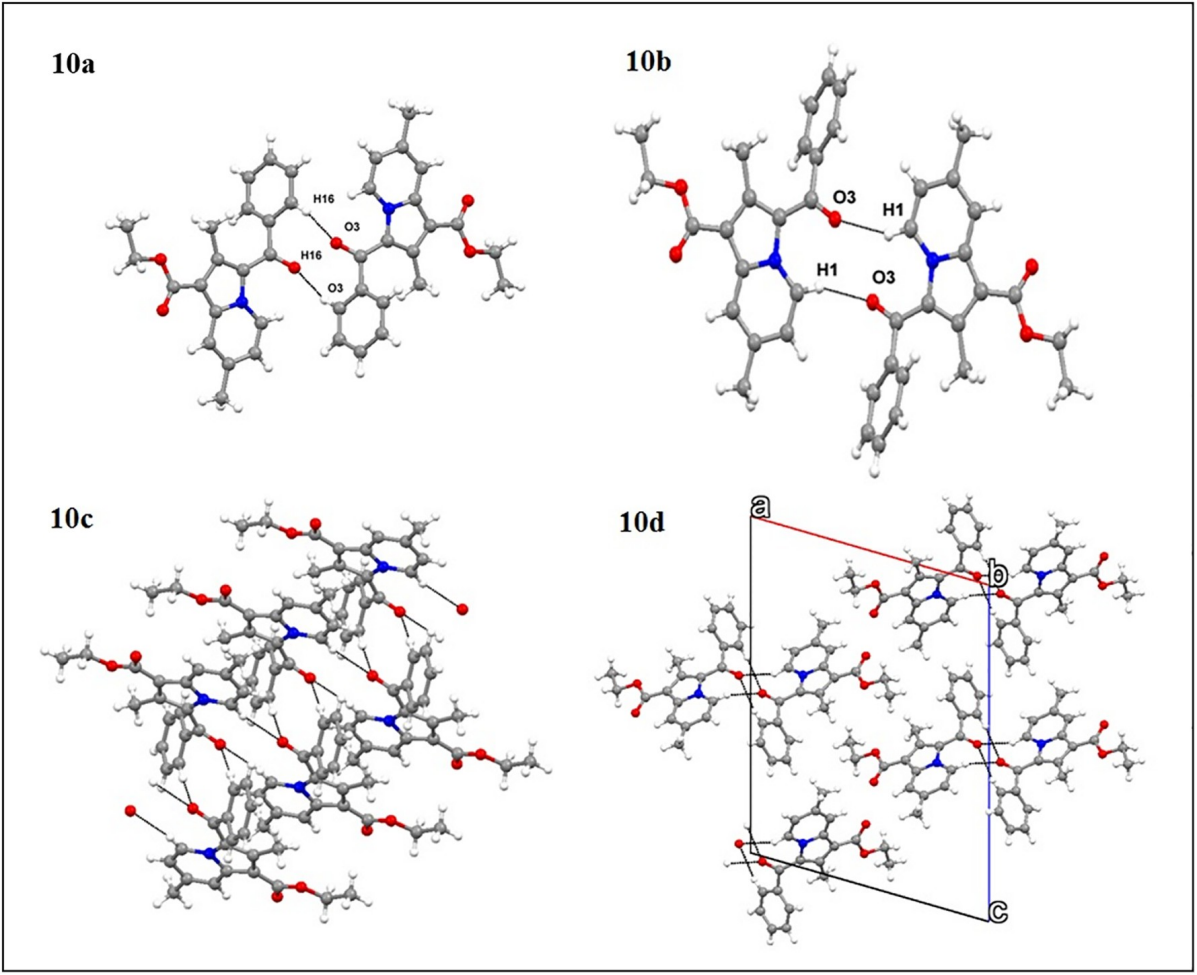
Crystallographic characterization of INZL. (10a) Dimer formation through C-H16⋯O3 hydrogen bonds. (10b) The molecules form pairwise C**–**H1⋯O3 hydrogen bonds; (10c) The bifurcated C**–**H⋯O weak hydrogen bonding pattern through two different dimers. (10d) Molecular assembly shown along the b-axis.

The crystal structure of **INLZ** prefers a monoclinic crystal system with a centrosymmetric space group (P 2_1_/c); it features one molecule within the asymmetric unit cell and four molecules in the unit cell. Fig 9 illustrates a thermal ellipsoid plot along with atom labeling, and adopts the conformation using intra-molecular C-H⋯O (C1-H1 …O3; 2.26 Å, 117°, and C4-H4 …O1 2.46 Å, 116°) hydrogen bonds. The crystal structure prefers a head-to-head pairwise arrangement using two different types of C-H⋯O hydrogen bonds (C16-H16⋯O3; 2.45 Å, 167°, and C1-H1⋯O3; 2.30 Å, 155°; Fig 10a and 10b). Further, the C-H⋯π (C13**–**H13⋯Cg_1_ [the centroid of the five-member ring of N1/C5/C6/C7/C8]; 2.93 Å) band π⋯π (Cg_1_⋯Cg_2_ [the centroid of the six-member ring of N1/C1/C2/C3/C4/C5]; 3.90 Å) stabilized its zig-zag pattern (as shown in Fig 10c) and along the b-axis of the unit cell (Fig 10d). The molecules share the same structure orientation as seen with the common functional group of carbonyl and ester; as a result, all molecules are expected to exhibit a similar conformation and supramolecular assembly as reported in this case.

## Conclusions

To better understand the interactions between differently designed inhibitors and NANAS, as well as to guide the future molecular design of a small-molecule NANAS inhibitor, a virtual screening process was conducted while considering the currently available structural features of both the binding site and its associated inhibitors. This study identified a novel designed molecule that fulfilled Lipinski’s rule of five for druggable compounds. INLZ shows better fitness results in docking studies when compared with the previously reported NmeNANAS enzyme inhibitors. Furthermore, our molecular modeling simulation indicated the involvement of the Arg314 amino acid in INLZ–NmeNANAS interactions, which served as an important residue at the AFPL domain of the catalytic site. Ethyl 3-benzoyl-2,7-dimethyl indolizine-1-carboxylate (INLZ) represents a novel scaffold of NmeNANAS enzyme inhibitors and opens the gate for a new class of inhibitors with distinctive structural features.

The effect of inhibition on the NmeNANAS enzyme is associated with a great degree of potential bactericidal activity against *N*. *meningitidis*, especially serotype B for particular strains that produced a sialic acid capsule and/or showed resistance to penicillin. In the presence of an activated immune system, the reported data indicate the absence of non-capsulated strains of *N*. *meningitidis* bacteria in meningitis patients’ blood. However, no reported studies have tested the reliability of these data. Finally, improving immune system reactivity warrants much-needed therapeutic synergy.

## Supporting information

S1 Fig FT-IR of ethyl 3-benzoyl-2,7-dimethylindolizine-1-carboxylate (INLZ). (TIF)

S2 Fig ^1^H-NMR of ethyl 3-benzoyl-2,7-dimethylindolizine-1-carboxylate (INLZ). (TIF)

S3 Fig ^13^C-NMR of ethyl 3-benzoyl-2,7-dimethylindolizine-1-carboxylate (INLZ). (TIF)

## Abbreviations

AFPL: Antifreeze protein-like
ESI: Electrospray ionization
FT-IR: Fourier-transform infrared spectroscopy
INLZ: Indolizine
KBr: Potassium bromide
LC-MS: Liquid chromatography–mass spectrometry
ManNAc: *N*-acetyl mannosamine
NANA: *N*-acetylneuraminic acid
NANAS: *N*-acetylneuraminic acid synthase
NmeNANAS: N. meningitidis NANAS
NMR: Nuclear magnetic resonance spectroscopy
PEP: Phosphoenolpyruvate
TLC: Thin Layer Chromatography
TMS: Tetramethylsilane
