# Supplementary figures and images for: Design, synthesis, and structural elucidation of novel NmeNANAS inhibitors for the treatment of meningococcal infection
Source: PLoS One. 2019 Oct 16;14(10):e0223413. doi: 10.1371/journal.pone.0223413 (PMC6795526; doi:10.1371/journal.pone.0223413)

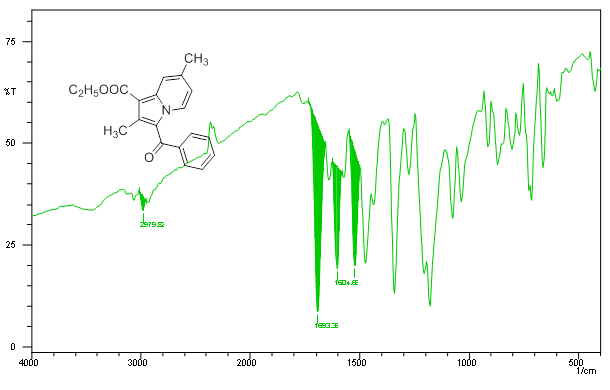

Supplement: S1 Fig — (TIF) [file pone.0223413.s001.tif]

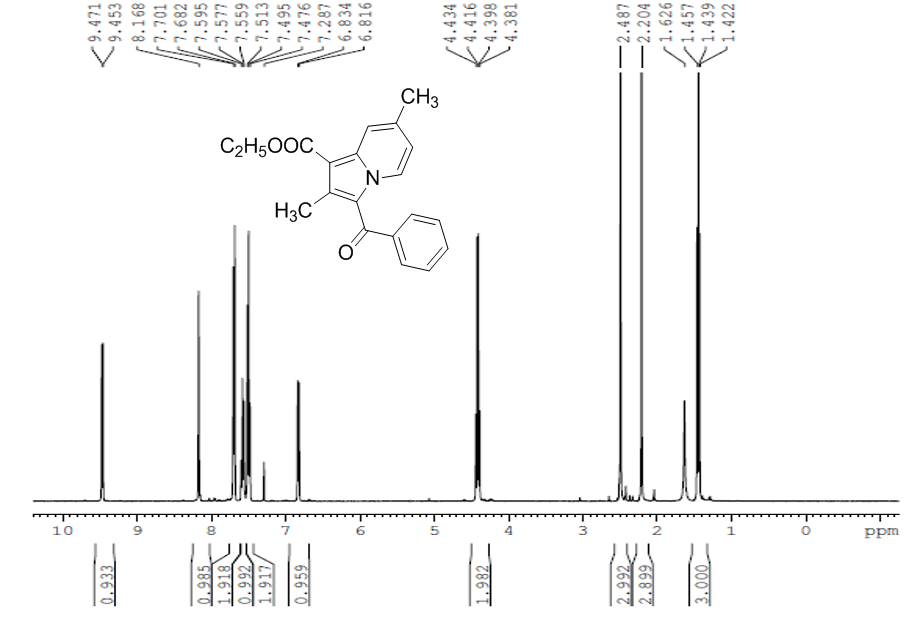

Supplement: S2 Fig — (TIF) [file pone.0223413.s002.tif]

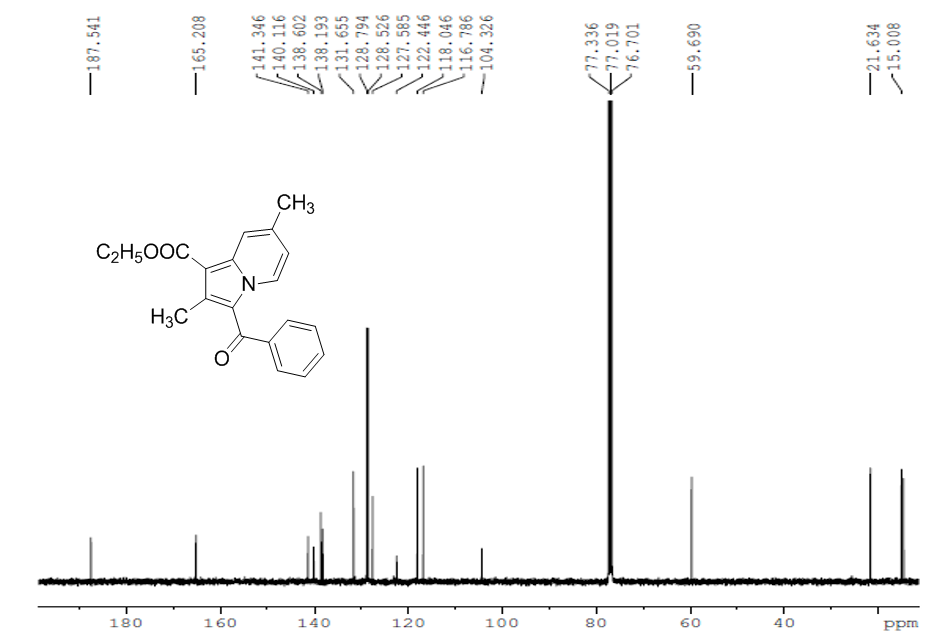

Supplement: S3 Fig — (TIF) [file pone.0223413.s003.tif]
